# Quantifying the level of difficulty to treat major depressive disorder with antidepressants: Treatment Resistance to Antidepressants Evaluation Scale

**DOI:** 10.1371/journal.pone.0227614

**Published:** 2020-01-14

**Authors:** Tzu-Yu Liu, Po-Hsiu Kuo, Mong-Liang Lu, Ming-Chyi Huang, Chun-Hsin Chen, Tzu-Hua Wu, Sabrina Wang, Wei-Chung Mao, Hsi-Chung Chen

**Affiliations:** 1 Department of Psychiatry, National Taiwan University Hospital, Taipei, Taiwan; 2 Graduate Institute of Epidemiology and Preventive Medicine, College of Public Health, National Taiwan University, Taipei, Taiwan; 3 Department of Psychiatry, Wan-Fang Hospital; Taipei & School of Medicine, College of Medicine, Taipei Medical University, Taipei, Taiwan; 4 Department of Psychiatry, Taipei City Hospital, Songde Branch, Taipei, Taiwan; 5 Department of Clinical Pharmacy, School of Pharmacy, College of Pharmacy, Taipei Medical University, Taipei, Taiwan; 6 Institute of Anatomy and Cell Biology, School of Medicine, National Yang-Ming University, Taiwan, Taipei, Taiwan; 7 Department of Psychiatry, Cheng-Hsin General Hospital, Taipei, Taiwan; Chiba Daigaku, JAPAN

## Abstract

**Background:**

The present study aimed to develop a new scale to evaluate the level of difficulty in treating major depressive disorder with antidepressants based on the lifetime treatment profile.

**Methods:**

In addition to evaluating the difficulty of treatment with antidepressants (A subscale), the Treatment Resistance to Antidepressants Evaluation Scale (TRADES) is comprised of a subscale to account for the attributes that compromise the efficacy of treatment (B subscale). One hundred and six participants aged 18 to 65 years with remitted major depressive disorder were enrolled. Eligible cases were those with at least 2 years from disease onset until the scoring date of the TRADES (the index date), with a complete treatment record. Various psychosocial and clinical features, such as neuroticism, harm avoidance, and utilization of psychiatric services, were used to validate the TRADES.

**Results:**

The mean duration of the course before and after the index date were 5.5 ± 3.5 and 3.1 ± 1.7 years, respectively. In a multiple regression analysis, the final total scores of the TRADES independently correlated with higher levels of neuroticism and harm avoidance. Total scores were also associated with a higher utilization of psychiatric outpatient and admission services before the index date. Furthermore, it is thought that total scores could predict a higher number of visits to psychiatric outpatient, emergency, and admission services following the index date.

**Conclusions:**

The TRADES has acceptable validity and could help to quantify the level of treatment difficulty with antidepressants in major depressive disorder.

## Introduction

Major depressive disorder (MDD) is a prevalent, but heterogeneous disorder [1]. Although the monoamine deficiency theory initiated the development of antidepressants, it also monopolized antidepressant classes and compounds. According to the Sequenced Treatment Alternatives to Relieve Depression study, the cumulative remission rate after four lines of treatment over 14 months was only 67% [2]. Thus, the homogeneous nature of antidepressants fails to provide adequate solutions for the heterogeneity of MDD. To enhance the etiological homogeneity of MDD, subtyping the level of treatment difficulty with antidepressants is fundamental.

Previous studies that aimed to subtype antidepressant treatment response in MDD can be classified into three types: phenotypic, endophenotypic, and genotypic levels [3]. At the phenotypic level, several factors have been consistently recognized to be associated with treatment response, such as disease course, gender, age, and psychiatric comorbidity [4]; however, there were no robust conclusions at the endophenotypic and genotypic levels [5, 6]. Since endophenotypic and genotypic studies require reliable phenotypes, inadequate validity of the phenotyping of treatment response to antidepressants may explain the inconsistent findings [7].

Antidepressants remain the treatment of choice for major depressive episodes and any biological intervention for MDD is often accompanied by at least one main-line antidepressant treatment; therefore, the construct of "difficult to treat with antidepressants" is similar to treatment resistant depression (TRD). Until 1995, TRD was dichotomously defined as an inadequate response that appears after two consecutive antidepressant treatments with adequate dosage and adequate duration (ADAD), but with antidepressants of different pharmacological classes [8]. After 1995, similar to the cancer staging system, the first staging model for TRD was developed [9]. When counting the number of failed trials of antidepressants, the subsequent multifaceted staging scales not only introduced various novel parameters to evaluate treatment response, but also discarded the traditional notion of different classes and hierarchical efficacy of antidepressants [8, 10]. The most recent staging instrument for the measurement of treatment resistance in MDD is the Maudsley Staging Model (MSM), which contains classical domains of treatment failure and is extended by including duration and baseline symptom severity of the index episode as novel parameters [11].

Although the MSM showed promise in predicting the risk and duration of a depressive episode during a follow-up period [12], recent literature suggests the inclusion of additional parameters to enhance the validity of measuring the level of treatment difficulty of MDD [4, 13]. For example, the use of sedatives, psychodynamic psychotherapy, physical and psychiatric comorbidities, and medication adherence may also influence the evaluation of antidepressant treatment response. Furthermore, current staging models only quantify the level of difficulty to treat a single episode, despite MDD being characterized by high recurrence and breakthrough rates [14, 15]. The course-dependent features of MDD should be considered as parameters when conceptualizing the level of treatment difficulty with antidepressants. Thus, concomitant consideration of the profile of the lifetime pattern of psychotropic use should better reflect the bona fides of the level of treatment difficulty, compared to the single episode approach. To the best of our knowledge, no such staging scale that comprises the entirety of the new perspectives has been reported in the literature.

Thus, the present study aims to develop a new, multi-dimensional, clinimetric instrument, the Treatment Resistance to Antidepressants Evaluation Scale (TRADES), to evaluate the level of treatment difficulty with antidepressants in MDD patients. Individuals in a remitted state with complete and longitudinal treatment medical records were included in the present study. By reviewing the lifetime disease course, information on treatment profiles were detailed with previously used and novel parameters. The performance of the TRADES was also examined using various psychosocial and clinical validators.

## Materials and methods

### Participants

The present study used a cohort that had been established by the Research Collaborating Group for New Insight, Strategy and Evaluation-Treatment-Resistant Depression Program. At two medical centers and one psychiatric hospital in Taiwan, participants were recruited from October, 2010 to April, 2016 who were aged 18 to 65 years, diagnosed with MDD based the Diagnostic and Statistical Manual of Mental Disorders, 4th edition, Text Revision criteria [16], and in a remitted state. Because MDD is a disease with high recurrence and breakthrough rates, TRADES was developed to evaluate the "***overall***" level of difficulty in treating "***each***" MDD episode with antidepressants, based on the lifetime treatment profile. Thus, after patients complete the treatment for each episode, then TRADES is allowed to capture the picture of overall difficulty of using antidepressants to treat an MDD patient. Furthermore, the effectiveness of using antidepressants as maintenance therapy is also regarded as a core component of "difficulty to treat with antidepressants.” Thus, the duration of stability in remitted status was included as a parameter in TRADES. Accordingly, only lifetime treatment history of remitted patients could be used.

To collect detailed information on medication use, number of visits to psychiatric outpatient units, and inpatient and emergency services use, a complete lifetime medical record throughout the disease course for each participant was required. The minimum duration that was required for an assessment with the TRADES was 2 years after the first onset of MDD. If the participants had discontinued treatment due to complete remission before the date of TRADES assessment (the index date), they were still eligible if the complete remission status had lasted, after the cessation of clinical interventions, until the index date. To examine the predictability of the TRADES after the index date, each participant had been followed for an additional period from their respective index dates to those ranging from November 2016 to February 2017. Patients with fragmented medical records or with comorbid organic brain syndromes, dementia, substance abuse, psychotic disorder, schizoaffective disorder, or bipolar affective disorder were excluded. Finally, a total of 106 individuals were enrolled. The present study was approved by the research ethics committees of the National Taiwan University Hospital, Wan-Fang Hospital and Taipei City Hospital, Songde Branch. Written informed consent was obtained from each participant and all methods were performed in accordance with the relevant guidelines and regulations.

### Design rationales for the TRADES

The TRADES was expected to provide a valid phenotypic reference that could be used in research for endophenotypes and genotypes that predict treatment response to antidepressants. The concept of treatment difficulty is broad and should comprise various domains, such as long-term stability, short-term severity, medication exposure experience, and non-pharmacological interventions (e.g., electroconvulsive therapy [ECT] and psychodynamic psychotherapy). Behavioral problems and comorbidities that may increase treatment difficulty should also be considered. Therefore, the TRADES was not established using psychometric methods, but has been conceptualized as a clinimetric instrument, which aims to integrate various parameters to capture the full picture of treatment difficulty with antidepressants in MDD [17]. Thus, two subscales, A and B, were designed to constitute the TRADES.

The A subscale provides a measurement of the difficulty of treating MDD with antidepressants and the B subscale provides a summary of the attributes that may bias the measurements of the A subscale. Final total scores are obtained by subtracting the scores of the B subscale from the scores of A subscale. The scoring of each item of the TRADES is based on the personal life chart of the patient, which is derived from the detailed medical chart review. In the present study, trained research assistants scored each life chart according to pre-determined scoring rules, which ensured within- and between-rater reliability. The TRADES and the details of the scoring rules are provided as S1 Table and S1 Text, respectively. According to the scoring rules, for eligible patients, the disease course duration between the date of first onset of MDD and the index date should be at least 2 years. This ensures that each patient has an equal opportunity to receive various sequential interventions, such as a shift between antidepressants with ADAD, combination, augmentation (e.g. anticonvulsants, lithium, antipsychotics, methylphenidate, or thyroxin), ECT, and psychodynamic psychotherapy. The details of the scoring points for each parameter are provided in S2 Text.

### Construct validators for the TRADES

#### Clinical validators

Because utilization of medical resources and pertinent costs have been reported to correlate with the treatment resistance of MDD, the use of psychiatric facilities was included as construct validators in the present study [18]. The number of psychiatric outpatient visits and hospital admissions of the participants, from MDD onset to the index date, were collected and used as convergent validators. Additionally, the number of psychiatric outpatient and emergency visits and admissions after the index date until the end of the follow-up period were also collected and used to examine the predictability of the TRADES. The present study hypothesized that higher TRADES scores would be associated with a higher utilization of various psychiatric facilities.

#### Psychosocial validators

At the index date, the Eysenck Personality Questionnaire-Revised (EPQ-R), the Tridimensional Personality Questionnaire (TPQ), and the Multidimensional Scale of Perceived Social Support (MSPSS) were administrated to evaluate the psychosocial characteristics of the participants. These psychosocial features were also used as convergent validators for the TRADES.

Individuals with specific personality traits are known to be vulnerable to MDD [19]. To assess the association between personality traits and the TRADES, we used two self-reported questionnaires, the EPQ-R [20] and TPQ [21]. We used the short-scale EPQ-R to measure neuroticism and extraversion (six items each). We also assessed novelty seeking and harm avoidance subscales using the short-form TPQ (12 items each), which showed good reliability and validity in previous studies [22]. We hypothesized that the TRADES score would be positively correlated with neuroticism and harm avoidance, but not related to extraversion or novelty seeking. Moreover, to assess the association between social support and the TRADES, the MSPSS (12 items) was used [23, 24]. High total scores indicate better subjective social support. The present study hypothesized that the TRADES score would be inversely related to the level of perceived social support.

### Statistical methods

The data were analyzed with SPSS version 12.0 (IBM; Armonk, NY, USA) and SAS version 9.4 (SAS Institute; Cary, NC, USA). In addition to descriptive analyses, an exploratory factor analysis was used to evaluate the internal consistency of the A subscale of the TRADES, which consisted of seven categorical variables. As no responses were recorded for the parameter "use of ECT", it was removed from the internal consistency analysis. An exploratory factor analysis was conducted using the principal factor estimation method “PROC FACTOR” in SAS with a polychoric correlation matrix among six variables. Eigenvalues greater than 1 were extracted [25]. The Kaiser-Meyer-Olkin measure for sampling adequacy was used to determine the appropriate sample size for conducting the factor analysis [26]. Finally, multiple linear regression analyses were conducted to examine the independent association between the parameters of the TRADES with the psychosocial and clinical validators. Stepwise linear regression analyses were also performed to select the most explanatory parameter of the A subscale that corresponded to each validator. The statistical significance level was set at *p* < 0.05.

## Results

Demographic and clinical characteristics of the participants are summarized in Table 1. A total of 106 individuals with MDD were enrolled (75.5% women). The mean age of the participants was 51.5 ± 13.2 years and the average age of MDD onset was 44.5 ± 13.6 years. The average scores of the A and B subscales of the TRADES were 5.3 ± 2.9 and 0.6 ± 0.7, respectively, and the average total score (subscale A-subscale B) was 4.6 ± 2.0. The average overall duration of follow-up was 9.8 ± 4.9 years. The average duration of follow-up before scoring the TRADES (the index date) was 5.5 ± 3.5 years, with a minimum of 2 years. The average duration of follow-up after the index date was 3.1 ± 1.7 years, with a minimum of 0.6 years. Table 2 depicts the distribution of the TRADES scores.

**Table 1:** Demographic and clinical characteristics of participants (n = 106).

	Mean ±SD
Age (years)	51.5±13.2
Age of first onset (years)	44.5± 13.6
Sex (n, %)	
Female	80 (75.5%)
Eysenck Personality Questionnaire	
Extraversion	3.7±2.8
Neuroticism	7.1±3.6
Tridimensional Personality Questionnaire	
Harm avoidance	10.3±3.4
Novelty-seeking	6.9±3.1
Multidimensional Scale of Perceived Social Support	54.1±12.5
	**Mean± SD, range**
Treatment Resistance to Antidepressants Evaluation Scale (TRADES)	
Total scores of the A subscale	5.3±2.9, 0–13
Total scores of the B subscale	0.6±0.7, 0–3
Total scores of the TRADES	4.6±2.0, 0–13
Follow-up duration (years)	
Overall duration	9.8±4.9, 2.6–22.3
Before the index date	5.5±3.5, 2.0–18.4
After the index date	3.1±1.7, 0.6–6.1
Clinical feature before the index date	
Frequencies of psychiatric admission	0.3±0.7, 0–4
Visits to psychiatric outpatient service	48.4±73.1, 7–529
Clinical feature after the index date	
Frequencies of psychiatric admission	0.1±0.5, 0–4
Visits to psychiatric outpatient service	15.2±18.5, 0–128
Visits to psychiatric emergency service	0.04±0.19, 0–1

**Table 2:** Scores distribution of the Treatment Resistance to Antidepressants Evaluation Scale (TRADES).

Dimension/ Parameter	Score	n (%)	Dimension/ Parameter	Score	n (%)
**A subscale**			**A3-4. Sedatives**		
**A1: Duration of stability**			Not used	0	4 (3.8)
Long (> 18 months)	0	50 (47.2)	Used <1/2 course	1	23 (21.7)
Mid-term (6–18 months)	1	24 (22.6)	Used ≥1/2 course	2	79 (74.5)
Short (≤ 6 months)	2	32 (30.2)	**A3-5. Psychotherapy**		
**A2: Symptom severity at index date**			Not used	0	95 (89.6)
Euthymic (BDI-II*: 0–16)	0	53 (50.0)	Used	1	11 (10.4)
Mild (BDI-II: 17–22)	1	11 (10.4)	**B subscale**		
Moderate (BDI-II:23–30)	2	20 (18.9)	**B1: Compliance**		
Severe (BDI-II:31–63)	3	22 (20.8)	Excellent	0	93 (87.7)
**A3: Treatment loads**			Good (more than 80% documented compliance)	1	13 (12.3)
** A3-1. Antidepressants**			Poor (fewer than 80% documented compliance)	2	0 (0.0)
Level 1: 1 medication	0	41 (38.7)	**B2: Psychiatric comorbidity**		
Level 2: 2 medications	1	43 (40.6)	Nil	0	64 (60.4)
Level 3: 3–4 medications	2	16 (15.1)	Axis I disorders	1	42 (39.6)
Level 4: ≥5 medications	3	6 (5.7)	Axis II disorders	2	0 (0.0)
** A3-2. Augmentation**			**B3: Chronic medical condition**		
Level 1: No augmentation	0	59 (55.7)	Nil	0	96 (90.6)
Level 2: 1 medication	1	30 (28.3)	1–2	1	10 (9.4)
Level 3: 2 medications	2	14 (13.2)	≥ 3	2	0 (0.0)
Level 4: ≥ 3 medications	3	3 (2.8)			
** A3-3. Electroconvulsive therapy**					
Not used	0	106 (100.0)			
Used	1	0 (0.0)			

*: BDI-II: Beck Depression Inventory-II

The internal consistency of the A subscale was examined using an exploratory factor analysis. The Kaiser-Meyer-Olkin value was 0.64, which indicated an acceptable sample size. Based on the scree plot and eigenvalue, a one-factor solution was selected. This factor had an eigenvalue of 1.94 and included six parameters in the A subscale, except for the use of ECT (question A3-3). This single factor model explained 91.6% of the variance. The factor loading of each parameter was delineated as follows: duration of stability (A1), 0.55; symptom severity (A2), 0.48; use of antidepressants with ADAD (A3-1), 0.76; use of augmentation agents (A3-2), 0.71; use of sedatives (A3-3), 0.54; and psychodynamic psychotherapy (A3-5), 0.19. Use of psychodynamic psychotherapy (A3-5) had the lowest factor loading.

The relationship between the TRADES and psychosocial validators is summarized in Table 3. In summary, the total scores of the A subscale were positively associated with neuroticism [b (se [standard error]) = 0.50 (0.12), *p* < 0.001] and harm avoidance [b (se) = 0.33 (0.13), *p* = 0.01], but negatively correlated with perceived social support [b (se) = ˗0.96 (0.45), p = 0.03]. In the B subscale, no single parameter was associated with psychosocial features; however, the total scores of the B subscale showed a positive correlation with novelty seeking [b (se) = 1.21 (0.51), *p* = 0.02] and a negative correlation with perceived social support [b (se) = ˗4.00 (2.00), *p* = 0.05]. Finally, the total scores of the TRADES were positively associated with the levels of neuroticism [b (se) = 0.49 (0.12), *p* < 0.001] and harm avoidance [b (se) = 0.32 (0.13), *p* = 0.01].

**Table 3:** Multiple regression analyses for the relationship between dimensions of TRADES and various psychosocial features*.

	Neuroticism		Extraversion		Harm-avoidance		Novelty-seeking		Perceived social support	
TRADES parameters^#^	b(se)	*p*	b(se)	*p*	b(se)	*p*	b(se)	*p*	b(se)	*p*
**A subscale**										
A1: Duration of stability	0.84 (0.44)	0.06	-0.12 (0.36)	0.75	0.76 (0.43)	0.08	-0.16 (0.39)	0.68	0.66 (1.51)	0.66
A2: Symptom severity at index date	1.13 (0.27)	<0.001	-0.01 (0.25)	0.99	0.87 (0.28)	0.003	0.02 (0.26)	0.94	-3.99 (0.93)	<0.001
A3: Treatment loads										
A3-1. Antidepressants	1.01 (0.44)	0.02	0.83 (0.36)	0.02	0.15 (0.45)	0.74	0.63 (0.39)	0.11	-0.29 (1.54)	0.85
A3-2. Augmentation	0.55 (0.49)	0.26	0.20 (0.40)	0.61	0.27 (0.48)	0.58	0.82 (0.42)	0.05	-0.71 (1.66)	0.67
A3-3. Electroconvulsive therapy	NA	NA	NA	NA	NA	NA	NA	NA	NA	NA
A3-4. Sedatives	1.33 (0.72)	0.07	0.31 (0.59)	0.60	0.70 (0.71)	0.33	1.01 (0.62)	0.08	-5.03 (2.52)	0.05
A3-5. Psychotherapy	2.19 (1.19)	0.07	-0.53 (0.98)	0.59	0.99 (1.18)	0.40	0.02 (1.04)	0.98	2.11 (4.07)	0.61
Total scores of A subscale	0.50 (0.12)	<0.001	0.08 (0.11)	0.47	0.33 (0.13)	0.01	0.13 (0.11)	0.27	-0.96 (0.45)	0.03
**B subscale**										
B1: Compliance	-0.08 (1.16)	0.95	-0.01 (0.25)	0.99	-0.21 (1.14)	0.86	0.02 (0.26)	0.94	-5.16 (3.87)	0.19
B2: Psychiatric comorbidity	-0.09 (0.76)	0.91	0.94 (0.62)	0.13	-0.68 (0.76)	0.37	1.13 (0.65)	0.09	-3.99 (2.56)	0.12
B3: Chronic medical condition	-1.00 (1.67)	0.55	-1.02 (1.36)	0.45	1.42 (1.63)	0.38	-0.90 (1.44)	0.53	-1.40 (0.10)	0.56
Total scores of B subscale	-0.27 (0.61)	0.65	0.10 (0.50)	0.83	-0.27 (0.60)	0.65	1.21 (0.51)	0.02	-4.00 (2.00)	0.05
Total (A-B) scores	0.49 (0.12)	<0.001	0.04 (0.11)	0.71	0.32 (0.13)	0.01	0.02 (0.11)	0.83	-0.67 (0.44)	0.13

*All models were controlled for sex, age of onset and duration of follow-up before the index date

^#^ TRADES: the Treatment Resistance to Antidepressants Evaluation Scale

Table 4 illustrates the relationship between the TRADES and various clinical features. The total scores of both the A subscale [b (se) = 5.22 (2.23), *p* = 0.02] and the TRADES [b (se) = 5.41 (2.16), *p* = 0.01] showed positive correlations with the number of visits to outpatient services. Additionally, the total scores of the A subscale [b (se) = 0.07 (0.02), *p* = 0.004] and the TRADES [b (se) = 0.07 (0.02), *p* = 0.002] showed positive correlations with the number of psychiatric admissions before the index date. The B subscale individual parameter or total scores did not correlate with the number of psychiatric outpatient visits or admission.

**Table 4:** Multiple regression analyses for the relationship between TRADES and lifetime clinical features by the index date*.

	Before the index date^#^				After the index date^ǂ^					
	Number of visits to outpatient service		Number of psychiatric admission		Number of visits to outpatient service		Number of psychiatric admission		Number of visits to psychiatric emergency	
	b(se)	*p*	b(se)	*p*	b(se)	*p*	b(se)	*p*	b(se)	*p*
**A subscale**										
A1: Duration of stability	9.84 (7.30)	0.18	0.09 (0.08)	0.28	3.43 (1.96)	0.08	0.14 (0.05)	0.01	0.04 (0.02)	0.09
A2: Symptom severity at the index date	14.74 (4.94)	0.004	0.06 (0.06)	0.30	4.04 (1.33)	0.003	0.07 (0.04)	0.06	0.03 (0.02)	0.04
A3: Treatment loads										
A3-1. Antidepressants	-0.61 (7.51)	0.94	0.20 (0.08)	0.01	0.83 (2.00)	0.68	0.03 (0.06)	0.59	0.05 (0.02)	0.03
A3-2. Augmentation	14.21 (7.98)	0.08	0.32 (0.08)	<0.001	1.47 (2.17)	0.50	0.13 (0.06)	0.03	0.06 (0.02)	0.02
A3-3. Electroconvulsive therapy	NA	NA	NA	NA	NA	NA	NA	NA	NA	NA
A3-4. Sedatives	6.35 (12.88)	0.62	0.01 (0.14)	0.95	6.19 (3.38)	0.07	0.07 (0.09)	0.49	0.05 (0.04)	0.21
A3-5. Psychotherapy	19.84 (21.37)	0.36	0.46 (0.23)	0.05	12.45 (5.58)	0.03	-0.11 (0.16)	0.50	0.16 (0.06)	0.01
**Total scores of A subscale**	5.22 (2.23)	0.02	0.07 (0.02)	0.004	1.65 (0.59)	0.01	0.04 (0.02)	0.01	0.02 (0.01)	0.001
**B subscale**										
B1: Compliance	-16.63 (18.99)	0.38	0.05 (0.20)	0.81	-2.67 (5.08)	0.60	-0.02 (0.14)	0.89	0.14 (0.06)	0.02
B2: Psychiatric comorbidity	-13.69 (12.62)	0.28	-0.17 (0.14)	0.22	-6.8 (0.33)	0.04	-0.10 (0.09)	0.30	-0.02 (0.04)	0.57
B3: Chronic medical condition	40.14 (21.96)	0.07	0.06 (0.24)	0.79	1.90 (5.99)	0.75	0.36 (0.16)	0.03	-0.04 (0.07)	0.61
**Total scores of B subscale**	-4.28 (9.06)	0.64	-0.06 (0.10)	0.54	-3.78 (2.39)	0.12	0.004 (0.07)	0.06	0.01 (0.03)	0.63
**Total (A-B) scores**	5.41 (2.16)	0.01	0.07 (0.02)	0.002	1.93 (0.56)	0.001	0.04 (0.02)	0.02	0.02 (0.01)	0.002

*TRADES: the Treatment Resistance to Antidepressants Evaluation Scale

^#^ Sex, age of onset and duration of follow-up before the index date were included as covariates

ǂ Sex, age of onset, duration of follow-up before the index date and duration of follow-up after the index date were included as covariates, respectively

The predictability of the TRADES, with respect to the utilization of psychiatric facilities after the index date, was also examined (Table 4). Higher total scores on the A subscale [b (se) = 1.65 (0.59), *p* = 0.01] and the TRADES [b (se) = 1.93 (0.56), *p* = 0.001] predicted more frequent visits to outpatient units. Overall, the total scores of the A subscale [b (se) = 0.04 (0.02), *p* = 0.01] and the TRADES [b(se) = 0.04 (0.02), *p* = 0.02] also predicted increased numbers of psychiatric admissions. Similar to the earlier observation, the total scores of the A subscale [b (se) = 0.02 (0.01), *p* = 0.001] and the TRADES [b (se) = 0.02 (0.01), *p* = 0.001] also predicted increased number of visits to psychiatric emergency units.

Table 5 illustrates the most explanatory parameters in the A subscale for each validator obtained using stepwise multiple linear regression analyses. Except for the number of antidepressants with ADAD used (A3-1), symptom severity at index date (A2) was the most explanatory parameter of all the psychosocial validators. With respect to the clinical validators before the index date, symptom severity (A2) and the number of augmentation agents used (A3-2) were the most explanatory parameters for the number of psychiatric outpatient and admission unit utilizations, respectively. However, when combined, symptom severity (A2) and psychodynamic psychotherapy use (A3-5) demonstrated the best predictability for the number of visits to outpatient service units after the index date. Duration of stability (A1) alone was most relevant to the number of psychiatric admissions after the index date. Finally, the number of augmentation agents used (A3-2) and the use of psychodynamic psychotherapy (A3-5) independently predicted the number of uses of psychiatric emergency services.

**Table 5:** Stepwise multiple regression analyses for most explanatory parameters for psychosocial and clinical features*.

	A1: duration of stability		A2: Symptom severity at the index date		A3: Treatment loads							
					A3-1: Antidepressants		A3-2: Augmentation		A3-4: Sedatives		A3-5: Psychotherapy	
	b(se)	*p*	b(se)	*p*	b(se)	*p*	b(se)	*p*	b(se)	*p*	b(se)	*p*
Psychosocial features*												
Neuroticism	-	-	1.13 (0.27)	<0.001	-	-	-	-	-	-	-	-
Extraversion	-	-	-	-	0.83 (0.36)	0.02	-	-	-	-	-	-
Harm-avoidance	-	-	0.87 (0.28)	0.003			-	-	-	-	-	-
Novelty-seeking	-	-			-	-	-	-	-	-	-	-
Perceived social support	-	-	-4.00 (0.93)	<0.001	-	-	-	-	-	-	-	-
Clinical feature before the index date*												
Number of visits to outpatient service	-	-	14.74 (4.94)	0.004	-	-	-	-	-	-	-	-
Number of psychiatric admission	-	-	-	-	-	-	0.32 (0.08)	<0.001	-	-	-	-
**Clinical feature after the index date**^**#**^												
Number of visits to outpatient service	-	-	3.94 (1.30)	0.003	-	-	-	-	-	-	11.89 (5.37)	0.03
Number of psychiatric admission	0.14 (0.05)	0.01	-	-	-	-	-	-	-	-	-	-
Number of visits to psychiatric emergency	-	-	-	-	-	-	0.05 (0.02)	0.04	-	-	0.14 (0.06)	0.03

* In each model, sex, age of onset and duration of follow-up before the index date were forcedly entered as covariates

^#^ In each model, sex, age of onset, duration of follow-up before and after the index date were forcedly entered as covariates, respectively

To determine an independent relationship between the TRADES and clinical validators, psychosocial validators that had been found to correlate with the TRADES (such as neuroticism, harm avoidance, and perceived social support), were included as covariates to remove potential confounding effects. In the analyses of clinical validators before the index date, after adding psychosocial factors into the previous models, the total scores of the TRADES continued to independently correlate with the number of psychiatric admissions [b (se) = 0.10 (0.03), *p* = 0.001]. However, the correlation with number of visits to outpatient services was brought down to a level of borderline significance [b (se) = 5.30 (2.79), *p* = 0.06]. After the index date, the total scores of the TRADES still independently predicted the number of visits to psychiatric outpatient [b (se) = 1.74 (0.72), *p* = 0.02], admission [b (se) = 0.04 (0.02), *p* = 0.05], and emergency [b (se) = 0.02 (0.01), *p* = 0.003] services.

## Discussion

Currently, the majority of the tools used to evaluate the level of treatment resistance in MDD are based on a single episode. In clinical practice, a patient demonstrating a good response to antidepressant treatment during a single episode may still experience relapse during the subacute or treatment maintenance phase. Similar to the strategy applied for a severe major depressive episode, therapists use combination or use augmentation agents upon failure of the intuitive intervention dose (i.e., escalate the dosage of the pre-existing antidepressant) [27]. However, a poor response to antidepressants within a single episode should promote the administration of various types of psychotropic agents. Although upon complete remission after a series of biological interventions and the completion of the course of maintenance therapy and a subsequently normal lifestyle without any medications, the patient is considered to be treatment resistant within the context of the single episode. Moreover, assuming that response to antidepressants is equal between each episode is speculative. Since MDD is characterized by recurrent and breakthrough episodes, measurement of the difficulty to treat with antidepressants based on only one episode appears inadequate to gauge the difficulty in a real-world setting. Compared to previous instruments, profiling treatment information throughout the lifetime disease course confers the advantages of the TRADES.

The present study used psychosocial and clinical features as convergent validators to determine the construct validity of the TRADES. As anticipated, the present study found that psychosocial features, namely neuroticism, harm avoidance, and perceived social support, were positively associated with the total scores of the A subscale; neuroticism and harm avoidance were also related to the final scores of the TRADES. Simultaneously, the present study used psychiatric outpatient, admission, and emergency services as clinical validators; higher final TRADES scores were associated with a higher frequency of use of psychiatric outpatient and admission services before the index date. The final TRADES scores also predicted higher use of the psychiatric outpatient, emergency, and admission services after the index date. These findings were also consistent with the expected results of our a priori hypothesis and further confirmed the construct validity of the TRADES.

In the present study, acute psychiatric admissions represent major depressive episodes with severe functional impairment; psychiatric emergencies may relate to an index major depressive episode or an acute unstable status. These two clinical validators reflect that an acute unstable status that warrants a higher intensity of care throughout life. In contrast, the frequency of use of outpatient services reflects the requirement of re-evaluation or stabilization of symptoms and is an indicator of long-term stability. This suggests that the TRADES comprises a full-spectrum relationship with symptomatic manifestations across the lifespan of patients with MDD.

The psychosocial validators adopted by the present study are risk factors for MDD [19]; therefore, individuals with these psychosocial features may be vulnerable to MDD because of their chronic reactive depressed mood, poor impulse control, and personality deviances. Under these circumstances, the TRADES scores may reflect emotional disturbances originating from maladaptive coping styles instead of a poor response to antidepressant treatment. It has been suggested that failure to remove the confounding effects from psychosocial characteristics would overestimate the magnitude of treatment difficulty [11]. In the present study, after controlling for neuroticism, harm avoidance, and perceived social support, the TRADES retained its association with clinical validators, which suggests the independent role of the TRADES in estimating the difficulty in treating MDD patients with antidepressants.

Using stepwise regression modeling, the present study examined the competitive capacity of the parameters of the A subscale to explain the variance of the validators. Firstly, for the duration of stability (A1), the trend of medication adjustment to estimate the duration of stable symptoms before the index date is used. This parameter reflects the confidence of the therapists on the overall stability of the patients. Our findings suggest that the duration of stability (A1), compared to the other parameters within treatment loads (A3), is the best predictor of future psychiatric admissions. Regardless of the severity of the previous episode, if the patient has been considerably stable, clinicians often lower the dosage and simplify the medication regimen. Thus, in continuing with the lifetime course evaluation, A1 balances the impact of a single or a severe episode in assessing treatment difficulty with antidepressants.

Secondly, considering a snapshot of symptom severity in the evaluation of the treatment response of antidepressants is a novel approach. In the MSM, the severity level of the initial depressive symptoms in the index episode is categorized as per the diagnostic criteria of the International Statistical Classification of Diseases and Related Health Problems, 10^th^ revision [11]. In contrast, for the A2 of the TRADES, the Beck Depression Inventory-II (BDI-II) is used to randomly sample the severity of depressive symptoms in the disease course of a remitted patient with MDD. Our findings demonstrate that symptom severity (A2) is most sensitive to the vulnerable psychosocial indicators (e.g., neuroticism, harm avoidance, and poor perceived social support) of the parameters of the A subscale. Interestingly, compared to the other parameters, A2 was most closely related to the frequency of use of outpatient services before and after the index date. Contrarily, A2 was not associated with psychiatric admissions when competing with other parameters. As the BDI-II, EPQ, TPQ, and perceived social support were collected at the index date, the overlap of underlying constructs among these psychometric instruments may explain the unique performance of A2 in the TRADES. In the present study, the psychosocial validators that showed an association with A2 reflect the vulnerability of the individuals to stress. Therefore, the reactive mood to stress may be transient and subsyndromal in severity and require intense outpatient care rather than an acute intervention with psychiatric admission.

In the stepwise regression model, of the various components of treatment loads (A3), the use of antidepressants with ADAD (A3-1) failed in effectively explaining any of the clinical validators. In the multiple linear regression analysis with the forced entry method, A3-1 was correlated with the number of psychiatric admissions before the index date and the number of emergency services used after the index date; however, these associations were undermined by those obtained in the stepwise regression analysis, namely, the use of augmentation agents (A3-2) and psychodynamic psychotherapy (A3-5). This finding is understandable and simply indicates the priority or choice of treatment in MDD. In clinical settings, augmentation or psychodynamic psychotherapy is not regarded as the first-line choice of treatment for a major depressive episode. For statistical considerations such as in our study, the use of augmentation agents and psychodynamic psychotherapy assume superiority over the use of antidepressants with ADAD in the stepwise regression analysis, because they are usually the consequence of failure of monotherapy, attempted with a series of antidepressants. As two failures with antidepressants with ADAD is the typically accepted cutoff indicating significant difficulty of treatment, which is generally followed by second-line interventions, our findings suggest that the scoring levels in A3-1 may be collapsed in future versions of the TRADES.

Lastly, psychotherapy (A3-5) was able to predict the number of visits to outpatient and emergency services after the index date but failed to predict psychiatric admissions after the index date. This finding suggests that A3-5 captures a subacute or chronic clinical scenario indicating that intensive outpatient care is sufficient to serve the clinical demand.

To summarize, the items selected for the A subscale were relevant as they appropriately constituted an individual construct for assessing the treatment difficulty with antidepressants. The parameters of the A subscale are unique with respect to their specific relationships with the various validators, which indicate the comprehension of item selection. These findings collectively support the content validity of the A subscale.

The method of deducting points from the core scale to correct for the impact of factors that may bias the measurement of treatment response has been reported in literature [28]. Our results illustrated that the total scores of the B subscale related to some of the validators, such as a higher level of novelty seeking, poorer perceived social support, and the number of psychiatric admissions after the index date, although with borderline statistical significance. This finding justified the introduction of the B subscale.

There are several limitations to the present study. Firstly, only 20.8% of the participants used more than two antidepressants with ADAD. It has been previously reported that 50% of MDD patients used two antidepressants with ADAD but failed to achieve remission [29]. It appears that the present study cohort included a group of patients with better treatment response. Similarly, none of the participants in the present study had been administered ECT. Although all the study sites conducted ECT in MDD patients, most patients requiring ECT had severe symptoms and were referred from regional hospitals to these study sites. This typically resulted in incomplete medical records of treatment experience, therefore, the inclusion criteria of the present study may result in the exclusion of patients with severe episodes. Due to these limitations, the generalizability of the TRADES should be exercised with caution. However, because ECT is a well-established indicator for TRD in the literature, it is retained in the current form of the TRADES to comprehensively conceptualize the construct of “difficulty to treat with antidepressants.” In the future, a more flexible use of the TRADES in big-data research may allow us to examine the contribution of ECT for the construct that the TRADES intends to describe. Secondly, in evaluating symptom stability, the trend of medication adjustment was used as a proxy for the long-term stability of depressive symptoms. However, it is uncertain whether changes in medication types or dosage are secondary to depressive symptoms or adverse side effects of medications. Thirdly, the TRADES assumed that all parameters had the same degree of impact on the evaluation of the treatment difficulty to antidepressants. Further study is required to evaluate the relative weights of each of the parameters.

## Conclusions

The present study developed a novel clinimetric instrument, the TRADES, to quantify the level of difficulty to treat MDD with antidepressants. In the future, the TRADES should be used in endophenotypic or genotypic research to examine its performance in serving as a reference for phenotypic classifications.

## Supporting information

S1 Table Treatment Resistance to Antidepressants Evaluation Scale (TRADES). (DOCX)

S1 Text Design rationales and scoring rules for the TRADES. (DOCX)

S2 Text Scoring points of each parameters for the TRADE. (DOCX)
