# Supplementary material for: Quantifying the level of difficulty to treat major depressive disorder with antidepressants: Treatment Resistance to Antidepressants Evaluation Scale
Source: PLoS One. 2020 Jan 14;15(1):e0227614. doi: 10.1371/journal.pone.0227614 (PMC6959551; doi:10.1371/journal.pone.0227614)
Supplement: S1 Table — (DOCX) [file pone.0227614.s001.docx]

| **Table S1. Treatment Resistance to Antidepressants Evaluation Scale (TRADES)** | | | | |
| --- | --- | --- | --- | --- |
| Dimension/ Parameter | Score | Dimension/ Parameter | Score |  |
| **A subscale** |  | **A3-4. Sedatives** |  |  |
| **A1: Duration of stability** |  | Not used | 0 |  |
| Long (> 18 months) | 0 | Used <1/2 course | 1 |  |
| Mid-term (6–18 months) | 1 | Used ≥1/2 course | 2 |  |
| Short (≤ 6 months) | 2 | **A3-5. Psychotherapy** |  |  |
| **A2: Symptom severity at index date** |  | Not used | 0 |  |
| Euthymic (BDI-II^*^: 0-16) | 0 | Used | 1 |  |
| Mild (BDI-II): 17-22 | 1 | **B subscale** |  |  |
| Moderate (BDI-II:23-30) | 2 | **B1: Compliance** |  |  |
| Severe (BDI-II:31-63) | 3 | Excellent | 0 |  |
| **A3: Treatment loads** |  | Good (more than 80% documented compliance) | 1 |  |
| **A3-1. Antidepressants** |  | Poor (fewer than 80% documented compliance) | 2 |  |
| Level 1: 1 medication | 0 | **B2: Psychiatric comorbidity** |  |  |
| Level 2: 2 medications | 1 | Nil | 0 |  |
| Level 3: 3–4 medications | 2 | Axis I disorders | 1 |  |
| Level 4: ≥5 medications | 3 | Axis II disorders | 2 |  |
| **A3-2. Augmentation** |  | **B3: Chronic medical condition** |  |  |
| Level 1: No augmentation | 0 | Nil | 0 |  |
| Level 2: 1 medication | 1 | 1-2 | 1 |  |
| Level 3: 2 medications | 2 | ≥ 3 | 2 |  |
| Level 4: ≥ 3 medications | 3 |  |  |  |
| **A3-3. Electroconvulsive therapy** |  |  |  |  |
| Not used | 0 |  |  |  |
| Used | 1 |  |  |  |
| ^*:^ BDI-II: Beck Depression Inventory-II | | | | |
