# Supplementary material for: Quantifying the level of difficulty to treat major depressive disorder with antidepressants: Treatment Resistance to Antidepressants Evaluation Scale
Source: PLoS One. 2020 Jan 14;15(1):e0227614. doi: 10.1371/journal.pone.0227614 (PMC6959551; doi:10.1371/journal.pone.0227614)
Supplement: S1 Text — (DOCX) [file pone.0227614.s002.docx]

**Design Rationales and Scoring Rules for the TRADES**

**(Treatment Resistance to Antidepressants Evaluation Scale)**

**Design Rationales of the TRADES**

***The A subscale***

The fundamental principle of the A subscale was as follows: monotherapy with only one antidepressant with adequate dosage and adequate duration (ADAD) and a long-term stable mood throughout the clinical course demonstrates patients with the lowest level of difficulty of treatment with antidepressants. Thus, parameters that were considered in the A subscale were duration of symptom stability (A1), symptom severity (A2), and treatment loads (A3).

Each component of the A subscale was designated to measure a specific domain of treatment difficulty with the use of antidepressants in major depressive disorder (MDD). Duration of stability (A1), by providing the trend of medication adjustment before the index date, reflected the long-term symptom stability. For participants who had been stable for a long period of time, medication adjustments were expected to be few and medication could be tapered in either dosage or medication type. In contrast, symptom severity (A2) was evaluated using the Beck Depression Inventory-II to represent a snapshot of the severity of depressive symptoms at a random time point in the disease course [1]. Thus, duration of stability (A1) and symptom severity (A2) depicted the degree of long-term and short-term disease stability, respectively. Treatment loads of antidepressants with ADAD (A3-1) and augmentation agents (A3-2) summarized the complete history of exposure to main-line psychotropic agents. The anchor points for the scoring levels were modified from the previous treatment resistant depression staging scales. The use of electroconvulsive therapy (ECT) (A3-3) is a classical indicator for severe or treatment resistant major depressive episodes [2]. It had been used as a parameter in several TRD scales, including the Thase & Rush Staging Model [3], European Staging Model [4], Massachusetts General Hospital Staging Model [5], and Maudsley Staging Model [6]. The use of sedatives (A3-4) indicates that the main-line psychotropic agents are inadequate in controlling the entire spectrum of psychopathology, particularly the subsyndromal anxiety/insomnia symptoms. Ideally, co-occurring subsyndromal anxiety/insomnia symptoms should remit with the alleviation of depressive symptoms. Heavy loads of sedatives suggest that the dysfunctional neurotransmitter systems associated with residual anxiety/insomnia symptoms may differ from that targeted by main-line psychotropics (e.g., the monoamine system). In Taiwan, long-term psychodynamic psychotherapy (A3-5) is often indicated in MDD after the treatment response to a series of biological interventions is unsatisfactory. Thus, A3-5 reflected the latent component of the treatment loads that could not be directly observed.

***The B subscale***

The B subscale is designed to evaluate the impact of factors that may compromise the effects of antidepressants. Parameters in the B subscale included compliance to medication (B1), psychiatric comorbidities (B2), and chronic medical conditions (B3).

Parameters in the B subscale may influence the treatment effect of antidepressants via the behavioral or biological pathways; therefore, their attributes should be deducted from the scores of the A subscale. If compliance (B1) is poor, the treatment difficulty with respect to antidepressants may be overestimated. Moreover, the bidirectional relationship between psychiatric (B2) and physical comorbidities (B3) with MDD may complicate the target of treatment and compromise the performance of antidepressants. Additionally, the side effect profiles of antidepressants or drug-drug interactions and chronic medical conditions (B3) may limit the selection of treatment of choice or optimal range of dosage.

**Scoring Rules for the TRADES**

**A: A subscale of the TRADES, B: B subscale of the TRADES**

1. All patients are scored based on their life charts, which should be prepared before scoring.

2. If the complete previous treatment history of a patient is not obtained from the medical records (e.g., if the patient had received prior treatment in more than one hospital), the patient can be assessed only if the information that is required for the TRADES can be obtained indirectly from the admission note or the patient’s report of the previous regimen. Particularly, the dates of specific treatments should be available. (Applies to all items of the A and B subscales)

3. The start point of the disease course is the time when MDD was diagnosed. (Applies to all items of the A and B subscales)

4. The index date is the date when the case was recruited and scored. (Applies to all items of the A and B subscales)

5. The diagnosis of MDD should be made at least 24 months before scoring (the index date). (A1)

6. The duration of stability is defined as the duration back tracked from the index date to the earliest date on which the main line treatment (including antidepressants and augmentation agents) was not increased in dosage or number of medications. (A1)

7. The symptom severity at the index date is measured with the Beck Depression Inventory-II. (A2)

8. Only antidepressants used in adequate dosage (according to the Ministry of Health and Welfare in Taiwan; suggested dosage for treating MDD) and for adequate duration (at least 2 weeks) are included. (A3-1)

9. For inclusion, the antidepressant should be approved and indicated for monotherapy of MDD by the Food and Drug Administration of Taiwan. When scoring, each antidepressant used counts as one, irrespective of the class or mechanism of action (similar to or different from other antidepressants) or method of use (single or combined). In the case of trazodone or tricyclic antidepressants, the dosage that satisfies one count should reach the therapeutic level for depression treatment. (A3-1)

10. The categories of augmentation agents include anticonvulsants, lithium, antipsychotics, methylphenidate, or thyroxin, irrespective of the dosage. Augmentation agents are included when they are used in combination with antidepressants for at least 4 weeks at any dosage. When scoring, it is not necessary to judge the primary indication for the augmentation agents, such as sedatives or an augmentation agent (e.g., low dosage quetiapine). If two or more augmentation agents are used in the same period, all of the augmentation agents are still counted. (A3-2)

11. Patients who received ECT, irrespective of the number of sessions or the duration of the final total seizure, are scored as treated with ECT. (A3-3)

12. The frequency of sedative use is estimated by the number of outpatient services that prescribed benzodiazepines or hypnotics. Low doses of trazodone before bedtime that do not reach therapeutic levels for treating MDD are designated as a hypnotic. (A3-4)

13. Psychotherapy is defined as long-term, individual psychodynamic psychotherapy. Supportive psychotherapy, re-educative psychotherapy, and intensive psychotherapy that are performed during regular visits to outpatient services should not be coded. (A3-5)

14. Non-compliance rate is calculated by (the number of times that non-compliance is clearly documented in the medical record)/(the total number of visits to outpatient services). Compliance rate is calculated by 100% minus the non-compliance rate. (B1)

15. Psychiatric comorbidity is coded according to the discharge note of the most recent psychiatric admission or the most recent medical record made during the outpatient visit prior to the index date. (B2)

16. A chronic medical condition is included only when the diagnosis has appeared consecutively for at least three times in the lifetime course before the index date. Only diseases that are listed on Charlson comorbidity index are coded. (B3)

17. The final total score of the TRADES is obtained by subtracting the total score of the B subscale from the total score of the A subscale. If the total score of the B subscale is greater than that of the A subscale, the data of the participant should be excluded from the analysis.

**References**

1. Beck AT, Steer RA, Ball R, Ranieri W. Comparison of Beck Depression Inventories -IA and -II in psychiatric outpatients. J Pers Assess. 1996;67(3):588-97. Epub 1996/12/01. doi: 10.1207/s15327752jpa6703_13. PubMed PMID: 8991972.

2. Folkerts HW, Michael N, Tolle R, Schonauer K, Mucke S, Schulze-Monking H. Electroconvulsive therapy vs. paroxetine in treatment-resistant depression -- a randomized study. Acta Psychiatr Scand. 1997;96(5):334-42. Epub 1997/12/12. doi: 10.1111/j.1600-0447.1997.tb09926.x. PubMed PMID: 9395150.

3. Thase ME, Rush AJ. When at first you don't succeed: sequential strategies for antidepressant nonresponders. J Clin Psychiatry. 1997;58 Suppl 13:23-9. Epub 1997/01/01. PubMed PMID: 9402916.

4. Souery D, Amsterdam J, de Montigny C, Lecrubier Y, Montgomery S, Lipp O, et al. Treatment resistant depression: methodological overview and operational criteria. Eur Neuropsychopharmacol. 1999;9(1-2):83-91. Epub 1999/03/19. doi: 10.1016/s0924-977x(98)00004-2. PubMed PMID: 10082232.

5. Fava M. Diagnosis and definition of treatment-resistant depression. Biol Psychiatry. 2003;53(8):649-59. Epub 2003/04/23. doi: 10.1016/s0006-3223(03)00231-2. PubMed PMID: 12706951.

6. Fekadu A, Wooderson S, Donaldson C, Markopoulou K, Masterson B, Poon L, et al. A multidimensional tool to quantify treatment resistance in depression: the Maudsley staging method. J Clin Psychiatry. 2009;70(2):177-84. Epub 2009/02/05. doi: 10.4088/jcp.08m04309. PubMed PMID: 19192471.
