# Supplementary material for: Quantifying the level of difficulty to treat major depressive disorder with antidepressants: Treatment Resistance to Antidepressants Evaluation Scale
Source: PLoS One. 2020 Jan 14;15(1):e0227614. doi: 10.1371/journal.pone.0227614 (PMC6959551; doi:10.1371/journal.pone.0227614)
Supplement: S2 Text — (DOCX) [file pone.0227614.s003.docx]

**Scoring Points of Each Parameter for the TRADES**

**(Treatment Resistance to Antidepressants Evaluation Scale)**

**A subscale of the TRADES**

1. Duration of symptom stability (A1): The duration was divided and scored as: shorter than 6 months, 2 points; between 6 to 18 months, 1 point; and duration more than 18 months, 0 points.
2. Symptom severity (A2): The severity was scored according to the cutoffs suggested in Beck Depression Inventory-II (BDI-II). A2 was scored as 0 with BDI-II: 0–16, 1 with BDI-II: 17–22, 2 with BDI-II: 23–30, and 3 with BDI-II: 31–63.
3. Treatment loads (A3): Treatment loads were classified into five components comprising: the number of antidepressants with adequate dosage and adequate duration (ADAD) used (A3-1), the number of augmentation agents used (A3-2), use of electroconvulsive therapy (ECT; A3-3), use of sedatives (A3-4), and use of psychodynamic psychotherapy (A3-5). These components were scored as per the treatment experience throughout the lifetime disease course, which spanned the duration from the first onset of major depressive disorder to the index date.
4. Antidepressants with ADAD (A3-1): A3-1 was scored based on the number of antidepressants with ADAD used as follows: 0 points, one antidepressant (level 1); 1 point, two antidepressants (level 2); 2 points, three to four antidepressants (level 3); and 3 points, ≥ five antidepressants (level 4).
5. Augmentation agents (A3-2): Four levels of A3-2 were defined. Level 1 (0 points) indicated no exposure to any augmentation agent throughout the disease course. Level 2 (1 point) meant only one augmentation agent had been prescribed. Level 3 (2 points) referred to two augmentation agents used. Finally, level 4 (3 points) implied that ≥ three augmentation agents had been used.
6. ECT (A3-3): Treatment with ECT was scored as 1 point and treatment without ECT was scored as 0 points.
7. Benzodiazepines/hypnotics (A3-4): The scores were dependent on the duration of exposure to sedatives in contrast with the total disease course. The three categories included: never used, 0 points; combined use for less than half of the entire course, 1 point; and combined use for more than half of the entire course, 2 points.
8. Psychotherapy (A3-5): Treatment with long-term, individual psychodynamic psychotherapy was scored as 1 point and treatment without was scored as 0 point.

**B subscale of the TRADES**

1. Compliance to medications (B1): B1 was categorized as excellent, good, and poor. Excellent (0 points) meant that the entire medical record before the index date did not indicate any problems with medication adherence. Good (1 point) indicated more than 80%, but not 100% recorded adherence to medications. Poor (2 points) implied less than 80% recorded compliance to medication.
2. Psychiatric comorbidity (B2): B2 consisted of three categories: no psychiatric comorbidities, 0 points; axis I disorders based on the Diagnostic and Statistical Manual of Mental Disorders, 4th edition, Text Revision (DSM-IV-TR), 1 point; and axis II disorders in the DSM-IV-TR, 2 points.
3. Chronic medical condition (B3): B3 consisted of three categories: no physical diseases, 0 points; one or two diseases, 1 point; and more than two diseases, 2 points.
